# Retinal waves in adaptive rewiring networks orchestrate convergence and divergence in the visual system

**DOI:** 10.1162/netn_a_00370

**Published:** 2024-10-01

**Authors:** Raúl Luna, Jia Li, Roman Bauer, Cees van Leeuwen

**Affiliations:** Department of Psychobiology and Methodology in Behavioural Sciences, Faculty of Psychology, Universidad Complutense de Madrid, Madrid, Spain; Institute of Optics, Spanish National Research Council (CSIC), Madrid, Spain; KU Leuven, Brain and Cognition, Leuven, Belgium; NICE Research Group, Computer Science Research Centre, University of Surrey, Guildford, UK; RPTU Kaiserslautern, Cognitive Science, Kaiserslautern, Germany

**Keywords:** Evolving networks, Retinal ganglion cells, Synaptic remodeling

## Abstract

Spontaneous retinal wave activity shaping the visual system is a complex neurodevelopmental phenomenon. Retinal ganglion cells are the hubs through which activity diverges throughout the visual system. We consider how these divergent hubs emerge, using an adaptively rewiring neural network model. Adaptive rewiring models show in a principled way how brains could achieve their complex topologies. Modular small-world structures with rich-club effects and circuits of convergent-divergent units emerge as networks evolve, driven by their own spontaneous activity. Arbitrary nodes of an initially random model network were designated as retinal ganglion cells. They were intermittently exposed to the retinal waveform, as the network evolved through adaptive rewiring. A significant proportion of these nodes developed into divergent hubs within the characteristic complex network architecture. The proportion depends parametrically on the wave incidence rate. Higher rates increase the likelihood of hub formation, while increasing the potential of ganglion cell death. In addition, direct neighbors of designated ganglion cells differentiate like amacrine cells. The divergence observed in ganglion cells resulted in enhanced convergence downstream, suggesting that retinal waves control the formation of convergence in the lateral geniculate nuclei. We conclude that retinal waves stochastically control the distribution of converging and diverging activity in evolving complex networks.

## Author Summary

Retinal waves consist of spontaneous neural activity that propagates across the retina during neural development. We simulate the intermittent spread of retinal waveforms originating from a designated node in an adaptively rewiring neural network model. Adaptive rewiring models simulate, in a highly abstracted manner, how brains may achieve their complex topologies during development. This way, we aim to uncover basic principles of neural maturation in the visual system. Namely, we seek to shed light onto how retinal waves might be responsible for the differentiation of immature neurons into specific cell types (e.g., retinal ganglion cells, amacrine cells) and how these waves shape the connectivity structure in the visual system.

## INTRODUCTION

The connectivity of the central nervous system is a work in progress. During development and learning, synapses are being added, removed, and remodeled in response to neural network activity. Although later changes are thought to be largely experience-dependent (e.g., Himmelberg et al., 2023), a prominent role is reserved for spontaneous neural activity, at least before birth and in early development (Katz & Shatz, 1996). Spontaneous activity as it occurs in the developing retina, cochlea, spinal cord, cerebellum, and hippocampus, among others, is patterned. It provides important signals for the development of neurons and their connections (Blankenship & Feller, 2010). A key example is retinal wave activity (Galli & Maffei, 1988; Luhmann, 2022; Shatz, 1996). It arises in the interaction of amacrine and retinal ganglion cells (Xu et al., 2016) and occurs spontaneously, including a period before any visual input is available (Maccione et al., 2014). This is because the ganglion cells precede the other retinal cells, in particular the photoreceptors, in development (D’Souza & Lang, 2020).

Retinal waves play a decisive role in the formation of the topographic map structure of the retina (Arroyo & Feller, 2016). As the sole retinal output units, retinal ganglion cells project the retinal waves onto V1 (Alexander et al., 2011), and to the visual system beyond (Ackman et al., 2012; Xu et al., 2016). The neurophysiological mechanisms of retinal wave propagation effects are complex, involving active signaling and diffusion within the neural architecture (Leybaert & Sanderson, 2012). Retinal waves occur in three stages of neural development, evoked by different forms of neurotransmission (Maccione et al., 2014; Xu et al., 2016).

A basic question, however, has remained underexplored: how the retinal ganglion cells could develop into retinal output hubs in the first place. This question is addressed here within the framework of adaptive rewiring models (Gong & van Leeuwen, 2003, 2004; Jarman et al., 2017; Li et al., 2023; Rentzeperis et al., 2022). Using highly simplified neural network models, adaptive rewiring aims to understand in a principled manner the emergence of complex large-scale structures of the brain, that is, modular small-world networks (Bullmore & Sporns, 2009) with rich-club effect (van den Heuvel & Sporns, 2011; Zamora-López et al., 2010). In adaptively rewiring networks with ongoing oscillatory (Gong & van Leeuwen, 2003, 2004) or spiking (Kwok et al., 2007) activity, the connectivity structure is updated continuously by establishing new connections between units that interact strongly, while underused ones are cut. This optimizes the communication efficiency of the networks, leading to modular small-world structures (Rubinov et al., 2009; van den Berg & van Leeuwen, 2004) with a rich-club effect (Hellrigel et al., 2019).

Subsequent adaptive rewiring studies have simplified modeling even further, by representing the flow of activity through random walks on a graph. This approach enabled closed-form specification of the activity flow in terms of network diffusion kernels (Jarman et al., 2017; Rentzeperis & van Leeuwen, 2021). A recent move towards greater neural realism takes into account that neural connections are preferentially formed locally (Antonello et al., 2022). This spatial preference was initially modeled by postulating wiring costs (Jarman et al., 2014). A large proportion of connections in the brain are formed by calcium signals percolating through gap junctions between spatially neighboring neurons (Hettiarachchi et al., 2010; Weiss et al., 2022). Therefore, later models introduced a special rewiring rule based on spatial proximity alone (Calvo Tapia et al., 2020; Li et al., 2023).

**Figure 1.**
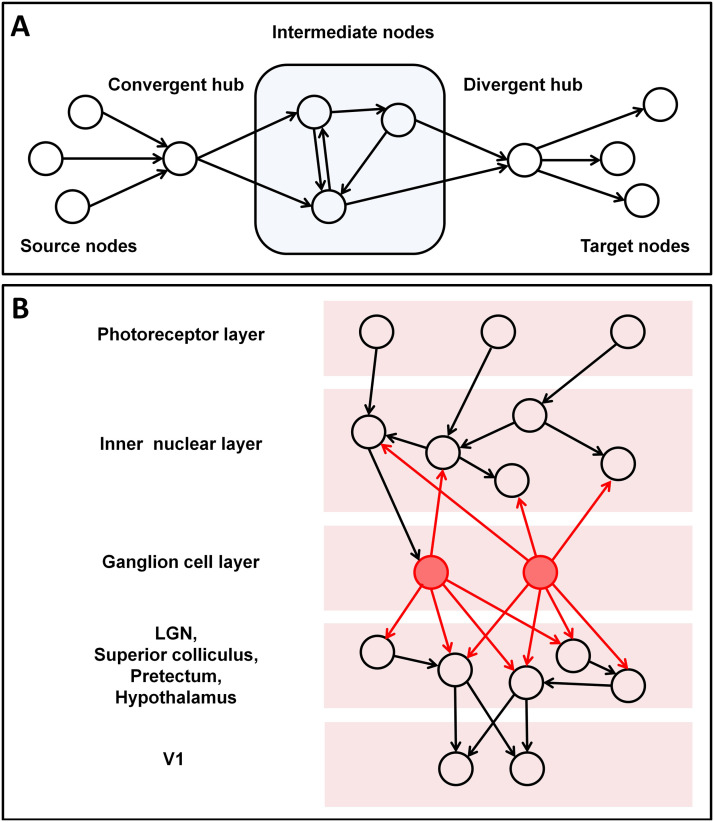
Schematic network representations. (A) From Li et al. (2023). Schema of a convergent-divergent unit. In a convergent-divergent unit, a convergent hub collects inputs and passes the information to a divergent hub through a subnetwork of intermediate nodes. The nodes sending information to the convergent hub are referred to as source nodes, and those receiving information from the divergent hub as target nodes. Note that typically the source and target nodes can show overlap; that is, a node can be both a source and a target node. (B) Schema of retinal circuitry. Photoreceptors feed their input into the rest of retinal cell types. Out of these, retinal ganglion cells (RGCs) act as divergent hubs that propagate their activity throughout the network, serving as input to other parts of the brain, such as the superior colliculus, the pretectum, the hypothalamus, and, importantly, the lateral geniculate nuclei (LGN), that connect with V1.

Following earlier work by Rentzeperis et al. (2022), Li et al. (2023) showed that adaptive and spatial rewiring jointly enable the formation of convergent-divergent units (Kumar et al., 2010; Shaw et al., 1982). Convergent-divergent units are network structures comprising one or more convergent hubs, which collect input from a range of local neurons and feed their signals to a relatively isolated set of units (a module), which in turn feeds a divergent hub, which broadcasts the output regionally or globally (Figure 1A). These units support neural signal flow and enable context-sensitive neural computation. Key examples of such units are the context-sensitive circuitry of V1: Activity converges on orientation-selective neurons in layers 2/3 that send their input to somatostatin (SOM) cells. These act as divergent hubs to broadcast their response back to the network (Adesnik et al., 2012; Niell & Scanziani, 2021), thereby enhancing orientation selectivity of V1 neurons (Song et al., 2020).

Divergent hubs also exist in the retina. Retinal ganglion cells (RGC) engage in reciprocal connections with amacrine cells in the inner nuclear layer and other retinal cells and are responsible in particular for the propagation of visual stimuli from both eyes to V1 as well as a number of other brain structures. Their axons leave the retina via the optic disk, joining to form the optic nerve en route to the optic chiasm, where about 60% of them cross over to the other side, while the rest remain uncrossed. From here, the fiber bundles, which now each contain RGC axons from both eyes, continue as the optic tract, to terminate in the superior colliculus, the pretectum, the hypothalamus, and the lateral geniculate nuclei (LGN) of the thalamus (Purves et al., 2001). The superior colliculus and the LGN, from which the visual signals ultimately reach V1 (Figure 1B), may be regarded as convergent hubs in downstream convergent-divergent units. In other words, even before V1 is reached, RGCs broadcast the visual signal to a number of brain regions. RGCs are responsible for distinct key visual computations, such as direction selectivity during motion processing, contrast detection, and orientation selectivity, among others (see Kerschensteiner, 2022, for a review).

### Ganglion Cells as Divergent Hubs

The role of RGC as divergent hubs is the main focus of our current study. The self-organizing networks of Li et al. (2023) lack facilities for receiving sensory input. Previous studies (Haqiqatkhah & van Leeuwen, 2022) showed that input systems could develop, and reach a degree of segregation in their entirety from the central network structure, without however destroying the integrity of the network. These studies did not offer suggestions on how retinal wave activity could constrain the evolving architecture of the visual system, though. We consider, in particular, whether retinal wave activity could play a decisive role in developing retinal ganglion cells into divergent hubs. We also consider whether this is achieved without hampering the developing complexity of the rest of the network, in particular, the emergence of convergent-divergent units.

### Model Strategy

For our model, we adopt the activity-based adaptive rewiring and spatial-proximity-based rewiring rules as proposed by Li et al. (2023). A third rule also used in Li et al. (2023), alignment, was not included here because its effect on network structure was found to be negligible. Adaptive rewiring in Li et al. (2023) is based on signal flow represented as network diffusion. Incoming diffusion for each node was determined according to a consensus criterion (Ren et al., 2007) and outgoing diffusion was determined according to advection (Chapman, 2015). A proportion of the times, adaptive rewiring was based on incoming flow, and a proportion on outgoing flow. In addition, a proportion of the times spatial rewiring was used, replacing a spatially distant connection with a spatially local one.

Initially random networks develop into complex networks according to iterative application of these rules. A subset of the nodes is designated to represent retinal ganglion cells. Retinal waves in ganglion cells are initiated in interaction with amacrine cells (Xu et al., 2016). We modeled this effect by supplying nodes designated as ganglion cells for a given proportion of times throughout the rewiring process with increased outgoing flow. We observe whether these nodes develop into divergent hubs. In addition, we consider the evolution of convergent-divergent units, and the participation of these designated nodes therein.

We find that over successive rewirings of an initially random network, as in Li et al. (2023), a complex network structure evolved that was spatially local, small-world, and modular, and that contained convergent-divergent units with both convergent and divergent hub nodes. A significant proportion of the nodes designated as ganglion cells developed into divergent hubs. The remaining hubs in the network developed spontaneously. This has the relevant implication that the formation of ganglia as divergent hubs in evolving complex networks can be stochastically controlled by the retinal waves themselves.

## METHOD

### Notation and Definitions

In accordance with Li et al. (2023), we define a directed graph (digraph) as the set *G* = (*V*, *E*, *W*), where *V* = {1, 2, …, *n*} represents the set of nodes, *E* ⊂ *V* × *V* denotes the set of ordered pairs of nodes, in which (*j*, *i*) ∈ *E* indicates directed edges from node *j* to *i* (*j* → *i*), and *W* = {*w*_*ij*_ : *i*, *j* ∈ *V*} represents the nodes’ weights. The adjacency matrix, *A* = [*A*_*ij*_]_*i*,*j*∈*V*_, with dimensions *n* × *n*, contains these weights, where *A*_*ij*_ = *w*_*ij*_, with *w*_*ij*_ > 0 when (*j*, *i*) ∈ *E* while the length of *j* → *i* is given by $\frac{1}{w_{ij}}$. When (*j*, *i*) ∉ *E*, *w*_*ij*_ = 0. |*V*| = *n* and |*E*| = *m*, respectively, denote the numbers of nodes and directed edges. Note that the weights are drawn from a probability distribution and will remain fixed, except when the retinal wave rule applies.

We define the in-links of a node *i* ∈ *V* as the set of edges directed towards *i*, and the out-links as the set of edges originating from *i*. The in-degree neighborhood of *i*, *N*_*in*_(*i*), comprises the tails of the in-links connected to *i*, while the remaining nodes, *V* − *N*_*in*_(*v*), are denoted as $N_{\text{in}}^{c}$(*v*). The in-degree of a node *i* is determined by the count of its in-links. Similarly, the out-degree neighborhood of *i*, denoted as *N*_*out*_(*i*), consists of the heads of the out-links emanating from *i*, while $N_{\text{out}}^{c}$(*v*) represents the remaining nodes. The out-degree of a node *i* is calculated as the number of its out-links. Considering an ordered pair of nodes (*u*, *v*), a directed walk from *u* to *v* is a sequential list of edges {(*i*_0_, *i*_1_), (*i*_1_, *i*_2_), …, (*i*_*K*−1_, *i*_*K*_) : *i*_0_ = *u*, *i*_*K*_ = *v*, (*i*_*k*−1_, *i*_*k*_) ∈ *E*} (Bender & Williamson, 2010). A directed walk is described as a directed path if all the vertices along it are distinct.

### Consensus and Advection Dynamics

The consensus and advection kernels represent the communication intensity between nodes, and are expressed as follows:

$$c\left(t\right)=e^{-L_{\text{in}}t}I_{n\times n}=e^{-L_{\text{in}}t},$$ (1)

and

$$\alpha \left(t\right)=e^{-L_{\text{out}}t}I_{n\times n}=e^{-L_{\text{out}}t},$$ (2)

where *c*(*t*) and *α*(*t*) are the consensus and advection kernels respectively. (See Appendix A, “Derivation of the consensus and advection kernels,” in the Supporting Information).

### Rewiring Rules

Our investigation focuses on analyzing the network’s structural changes through iterative rewiring of its edges. During each iteration, a node *v* ∈ *V* is randomly chosen for rewiring. If we are to rewire one of the in-links from *v*, an existing edge (*k*, *v*) ∈ *E* will be removed, and a new edge (*l*, *v*) ∉ *E* will be added, connecting *v* to a node *l* that was not previously linked to *v*. Similarly, when rewiring an out-link of *v*, we replace an existing edge (*v*, *k*) ∈ *E* with a new edge (*v*, *l*) ∉ *E*. To determine the selection of nodes *k* and *l* during each rewiring step, we employ one of the following principles, each with a fixed probability: the adaptive rewiring principle or the proximity-based principle.

#### Adaptive rewiring.

Adaptive rewiring implies that an underutilized connection is eliminated while a new connection is established between two previously unconnected nodes that exhibit the most intense flow between them, considering all indirect paths. A variety of network topologies emerge when rewiring the in-degree neighborhood using the consensus algorithm or rewiring the out-degree neighborhood using the advection algorithm (Rentzeperis et al., 2022). When rewiring in-links we employ the consensus kernel, and rewiring of out-links uses the advection kernel to estimate the communication intensity for rewiring the nodes.

When rewiring an in-link of node *v*, *k* is the node in *N*_*in*_(*v*) where (*k*, *v*) has the lowest value in the consensus kernel, denoted as *k* = *argmin*_*u*∈*N*_*in*_(*v*)_{*c*(*t*)_*vu*_}. Similarly, *l* is the node in $N_{\text{in}}^{c}$(*v*) such that (*v*, *l*) has the highest value in the consensus kernel, expressed as *l* = ${\text{argmax}}_{u\in N_{\text{in}}^{c}\left(v\right)}${*c*(*t*)_*vu*_}. When an out-link is rewired, *k* is the node in *N*_*out*_(*v*) such that (*v*, *k*) has the lowest value in the advection kernel, indicated by *k* = *argmin*_*u*∈*N*_*out*_(*v*)_{*a*(*t*)_*uv*_}. Correspondingly, *l* is the node in $N_{\text{out}}^{c}$(*v*) that makes (*l*, *v*) have the highest value in the advection kernel, and is represented as *l* = ${\text{argmax}}_{u\in N_{\text{out}}^{c}\left(v\right)}${*a*(*t*)_*uv*_}. Whether rewiring an in-link or an out-link of node *v*, *l* is the node chosen for rewiring in both cases.

Both the consensus and the advection kernels incorporate a time variable, *t*, set to a predetermined parameter known as the rewiring rate *τ*. This parameter signifies the duration between successive rewiring iterations (*τ* adopted a fixed value of 1 during our simulations).

##### Retinal wave rule.

This rule is a special case of adaptive rewiring. The otherwise constant out-link weights of a node *c*, to which we refer as *wave initiator* node, are boosted by adding a constant value, *B*, to their weights. A fixed value of *B* = 1 was adopted for our simulations. The boost represents the event that node *c* is releasing connection-forming Ca^2+^ in response to retinal wave activity. The weights from the entire network are then renormalized by dividing their values by the sum of all the weights. Next, rewiring takes place according to the adaptive rewiring rule. Afterward, all the weights, including those of node *c*, are reset to their original values, prior to adding the boost.

#### Proximity-based rewiring.

For this, we assume the digraph to be embedded within a two-dimensional Euclidean space. Each node *i* is denoted as ${\vec{x}}_{i}$ ∈ *R*^2^. In this space, proximity-based rewiring means that the longest connection is removed and substituted with the spatially shortest link between two nodes that were previously unconnected. The distance between node *i* and node *j* is calculated using *d*_*ij*_ = ∨${\vec{x}}_{i}-{\vec{x}}_{j}$∨, where ∨ · ∨ represents the Euclidean distance. When rewiring an in-link of node *v*, *k* is the node in *N*_*in*_(*v*) that has the greatest distance from *v*; that is, *k* = *argmax*_*u*∈*N*_*in*_(*v*)_{*d*_*uv*_}. Similarly, *l* is the node in $N_{\text{in}}^{c}\left(v\right)$ that has the shortest distance from *v*: $l={\text{argmin}}_{u\in N_{\text{in}}^{c}\left(v\right)}\left\{d_{uv}\right\}$. Conversely, when rewiring an out-link of node *v*, *k* is *argmax*_*u*∈*N*_*out*_(*v*)_{*d*_*vu*_}, and *l* is ${\text{argmin}}_{u\in N_{\text{out}}^{c}\left(v\right)}\left\{d_{vu}\right\}$.

### Rewiring Algorithm

For simplicity, the rewiring process maintains a constant number of nodes and edges within the networks. Other possibilities such as growing networks (Gong & van Leeuwen, 2003) and pruning networks (van den Berg et al., 2012) have also been investigated. To initiate the rewiring process, a random directed network *D* = (*V*, *E*, *W*) is established, with a given number of nodes, *n*, and edges, *m*. The *m* edges are assigned to node pairs, selected randomly without replacement from all possible *n*(*n* − 1) node pairs. Weights drawn from a normal probability distribution, *N*(1, 0.25^2^), are assigned at random to the edges. In the extremely unlikely event that a negative weight is sampled (3.17 × 10^−5^ probability), it is set to 0.05.

The iterative rewiring process follows as outlined below:

- *Step 1*: Randomly choose a node *v* ∈ *V* such that its in-degree is not zero and not equal to *n* − 1. Alternatively, choose a random node *v* ∈ *V* with an out-degree that is not zero and not equal to *n* − 1.
- *Step 2*: Determine whether to rewire an in-link or an out-link of *v* based on a probability value *p*_*in*_. If *p*_*in*_ is set to 1, only in-links are subject to rewiring, resulting in an iterative process known as in-link rewiring. Conversely, if *p*_*in*_ is set to 0, the iterative process is referred to as out-link rewiring (*p*_*in*_ adopted a fixed value of 0.5 during our simulations).
- *Step 3*: Depending on the outcome of Step 2, select a random in-link or out-link of *v* and rewire it using one of the following rewiring rules: adaptive rewiring, retinal wave rule (a special case of adaptive rewiring), or proximity-based rewiring. The probabilities of selecting each rule are denoted as *p*_*adaptive*_, *p*_*wave*_, and *p*_*proximity*_, respectively.
- *Step 4*: Repeat Steps 1 to 3 until a total of *M* edges have been rewired.

Network evolution was tested for different probabilities of *p*_*adaptive*_, *p*_*wave*_, and *p*_*proximity*_. For each set probability, 150 initially random networks were evolved throughout 4,000 iterations. Their resulting structure was analyzed using measures of graph topology, and the results were averaged across the 150 networks. Parameter *p*_*in*_ (i.e., the probability that either an in-link or an out-link is rewired) was set to a value of 0.5; *τ* (i.e., the duration between successive rewiring iterations) was fixed to 1; and *B* (i.e., strengthening of the out-link weights of a wave initiator node during wave occurrence) had a value of 1.

The following measures were used:

#### Modularity.

Modularity gauges the degree to which a network can be divided into distinct communities. In the context of a weighted digraph, we adopt the modularity definition proposed by Arenas et al. (2007):

$$Q=\frac{1}{2w}\sum_{i}\sum_{j}\left(w_{ij}-\frac{w_{i}^{\text{in}}w_{j}^{\text{out}}}{2w}\right)\delta \left(C_{i},C_{j}\right),$$ (3)

where *w* = ∑_*i*_∑_*j*_ *w*_*ji*_, $w_{i}^{\text{in}}=\sum_{j}w_{ji}$, $w_{i}^{\text{out}}=\sum_{i}w_{ji}$, *δ*(*C*_*i*_, *C*_*j*_) designates the Kronecker delta function, and *C*_*i*_ denotes to which community node *i* belongs. We use the algorithm by Leicht and Newman (2008) to find these communities.

#### Average efficiency.

The average efficiency metric measures how effectively information is transmitted across a network. It is calculated as the average of the reciprocals of the shortest directed path lengths between all pairs of nodes (Latora & Marchiori, 2001). Despite the networks being spatially embedded, we focus on assessing network efficiency concerning its connectivity structure and weights. We use the topological distance, *l*_*ij*_ = $\frac{1}{w_{ij}}$, which serves as an indicator of transmission complexity (Opsahl et al., 2010). At the neuronal level, a stronger synapse (large *w*_*ij*_) facilitates the easier transmission of electric nerve impulses between two neurons, resulting in smaller values of *l*_*ij*_.

For a given pair of nodes (*u*, *v*), a directed walk from *u* to *v* consists of an ordered sequence of edges {(*i*_0_, *i*_1_), (*i*_1_, *i*_2_), …, (*i*_*K*−1_, *i*_*K*_) : *i*_0_ = *u*, *i*_*K*_ = *v*, (*i*_*K*−1_, *i*_*K*_) ∈ *E*} (Bender & Williamson, 2010). A directed walk is a directed path when all its vertices are different. Average efficiency is defined as follows:

$$E=\frac{1}{n\left(n-1\right)}\sum_{i\neq j\in v}\frac{1}{l_{ij}},$$ (4)

where *l*_*ij*_ represents the length of the shortest directed path from node *i* to node *j*, signifying the most straightforward transmission route between the two nodes. In those cases where there is no transmission route from *i* to *j*, *l*_*ij*_ = ∞.

#### Number of connected node pairs.

A connectedness measure for a directed graph (digraph) is based on ordered pairs, (*i*, *j*), which are considered connected if there exists a directed path from node *i* to node *j*. The count of such connected node pairs serves as an indicator of the level of information exchange within the digraph. The upper limit for the number of connected node pairs is *n*^2^, and it occurs when every node can transmit information to any other node, including itself. We use this metric to quantify the degree of connectedness within a given digraph.

#### Convergent and divergent hubs, convergent-divergent units.

We classify nodes as convergent hubs if they have at least one out-link and a number of in-links that surpass a given threshold. These hubs serve as a substrate to collect dispersed information. Conversely, divergent hubs are nodes that possess at least one in-link and a number of out-links that exceed a predefined threshold. These hubs serve to disseminate information widely. We set the threshold for both convergent and divergent hubs to the value of 15 in all evaluations. We consider a convergent-divergent unit to be formed if a convergent and divergent hub are linked by one or more directed paths.

## RESULTS

### Modularity, Average Efficiency, and Connectedness Are Preserved in Networks With Retinal Waves

**Figure 2.**
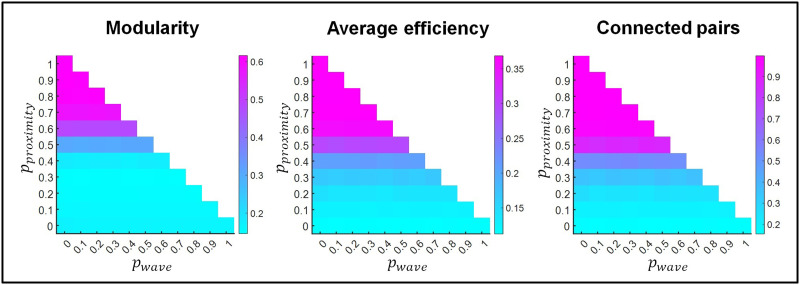
Modularity index (left), average efficiency (middle), and proportion of connected node pairs (right) of the networks resulting from rewiring. Results are shown as a function of the probability of proximity-based rewiring and retinal wave-based rewiring. The remaining probability (i.e., when the sum of the probabilities for proximity-based and retinal wave-based rewiring does not reach 1) corresponds to adaptive rewiring without retinal wave occurrence. For each combination of rewiring principle probabilities (e.g., *p*_*proximity*_ = 0.5, *p*_*wave*_ = 0.2, *p*_*adaptive*_ = 0.3), results are obtained by averaging over 150 networks that evolve from 150 initially random networks.

We assessed the average degree of modularity, efficiency, and connectedness in the resulting networks after 4,000 iterations. This number roughly represents the period of the first three postnatal weeks, in which synapses develop in the mouse retina (Hoon et al., 2014). The modularity indices in Figure 2 (left) show the extent to which the networks have a modular structure. Modularity increased with the proportion of proximity-based rewiring. Average efficiency and connectedness (Figure 2, middle and right, respectively) showed similar trends, suggesting that network efficiency is mostly related to its degree of connectedness. The occurrence of retinal waves did not interfere with the emergence of modular, efficient, and connected networks.

### Increased Number of Out-Links in Retinal Wave Initiator Nodes

**Figure 3.**
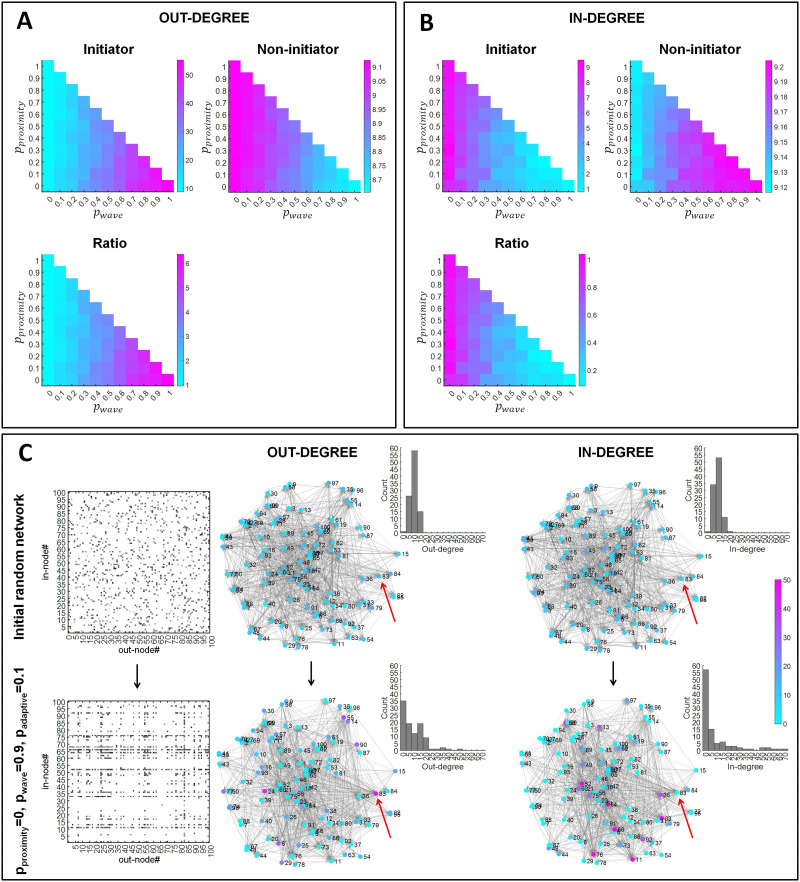
Out-link and in-link connectivity of retinal wave initiator and non-initiator nodes. (A, B) The left upper panels represent the average number of out-connections (A) and in-connections (B) in retinal wave initiator nodes. The right upper panels represent the same for non-initiator nodes. Note that the vertical scales differ between left and right panels within the top cells. The lower panels represent the ratio between the two upper panels, indicating the probability regions for the different rewiring principles where retinal wave initiator nodes most consistently develop a significant number of out-connections (A) and in-connections (B). Results are shown as a function of the probability of proximity-based rewiring and retinal wave-based rewiring. The remaining probability (i.e., when the sum of the probabilities for proximity-based and retinal wave-based rewiring does not reach 1) corresponds to adaptive rewiring without retinal wave occurrence. For each combination of rewiring rule probabilities (e.g., *p*_*proximity*_ = 0.5, *p*_*wave*_ = 0.2, *p*_*adaptive*_ = 0.3), results are obtained by averaging over 150 networks that evolve from 150 initially random networks. (C) Example network structure of an initially random network (upper plots) and a network after rewiring using *p*_*proximity*_ = 0, *p*_*wave*_ = 0.9, *p*_*adaptive*_ = 0.1 (lower plots). The leftmost plots show each of the adjacency matrices, indicating the out-connectivity and in-connectivity of each node, according to its identifier number (node#). The plots on their right show the spatially embedded networks. The numbers next to the nodes indicate the node identifier numbers, and the number of out-connections (OUT-DEGREE) and in-connections (IN-DEGREE) of each is expressed according to the color bar. The retinal wave initiator is Node 83 (indicated by a red arrow). For each network, the histograms depict the distributions of, respectively, the nodes’ in- and out-degree.

We investigated to what extent retinal wave initiator nodes developed into divergent hubs. Their number of out-links was compared with that of the other, non-initiator, nodes. By computing the ratio of their out-links, we obtained a metric of the consistency of wave initiator out-link development. The consistency was found to increase with an increasing proportion of retinal wave-based rewiring. For the non-initiator nodes, a minor tendency in the opposite direction was observed (i.e., the more seldom the waves, the larger the number of out-connections in non-initiator nodes; see Figure 3A). Indices analogous to those in Figure 3A were calculated for in-links (Figure 3B). Wave initiator nodes developed a lower number of in-links with larger proportions of retinal wave-based rewiring, whereas for non-initiator nodes in-link connectivity showed there was a minor tendency in the opposite direction.

Figure 3C (left) illustrates how an initially random network evolves with a high probability of wave-based rewiring (*p*_*wave*_ = 0.9). The wave initiator node (Node 83) develops the largest number of out-links among the nodes. Figure 3C (right) shows the in-link connectivity for the same network as in Figure 3C (left). The wave initiator node does not develop a large number of in-links. The in- and out-degree histograms in Figure 3C evidence the changes in network connectivity structure as a result of rewiring. See Figure S1 in the Supporting Information for more examples of network topologies resulting from different probabilities for *p*_*proximity*_, *p*_*wave*_, and *p*_*adaptive*_.

**Figure 4.**
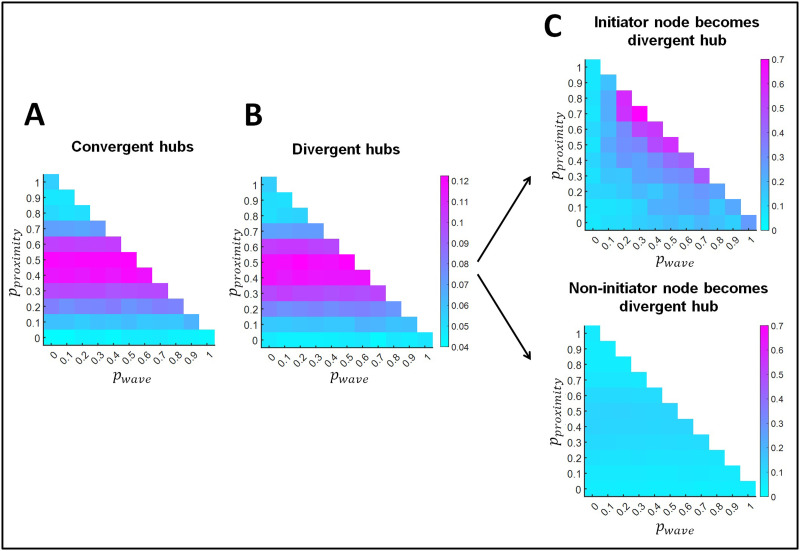
Formation of convergent and divergent hubs. (A) Proportion of convergent hubs. (B) Proportion of divergent hubs. (C) Upper panel: Proportion of network instances where retinal wave initiator nodes become a divergent hub. Lower panel: Out of 1,000 random selections of nodes that are not retinal wave initiators, proportion of times that these are a divergent hub. Results are shown as a function of the probability of proximity-based rewiring and retinal wave-based rewiring. The remaining probability (i.e., when the sum of the probabilities for proximity-based and retinal wave-based rewiring does not reach 1) corresponds to adaptive rewiring without retinal wave occurrence. For each combination of rewiring rule probabilities (e.g., *p*_*proximity*_ = 0.5, *p*_*wave*_ = 0.2, *p*_*adaptive*_ = 0.3), results are obtained by averaging over 150 networks that evolve from 150 initially random networks.

An important question is whether retinal waves interfere with the formation of convergent-divergent units. We first show the proportion of convergent and divergent hubs that exist in the networks resulting from rewiring (Figures 4A and 4B, respectively). Both convergent and divergent hubs most likely formed with intermediate proximity-based rewiring probabilities (i.e., in the range 0.4–0.5). In Figure 3A we show that for the largest wave-based rewiring probabilities, initiator nodes consistently developed the largest number of out-links. However, in these cases, initiator nodes also received the smallest number of in-links (see Figure 3B). To qualify as a divergent hub, a node needs to have an elevated number of out-links, but also at least one in-link. This criterion was rarely met when wave-based rewiring had the largest probability, and thus retinal wave initiator nodes did not often become divergent hubs in this case. For this reason, the greatest likelihood of finding initiator nodes developing into divergent hubs was obtained in the intermediate regime. Figure 4C (upper panel) shows that initiator nodes very likely became divergent hubs (i.e., probabilities around 0.6) when proximity-based rewiring was in the probability range 0.5–0.8 and wave-based rewiring was in the range 0.2–0.5.

On the other hand, when we randomly selected 1,000 non-initiator nodes and tested whether they were divergent hubs, we evidenced a much lower probability than that of initiator nodes for becoming divergent hubs (Figure 4C, lower panel). This probability was maximal when proximity-based rewiring was in the range 0.4–0.5.

**Figure 5.**
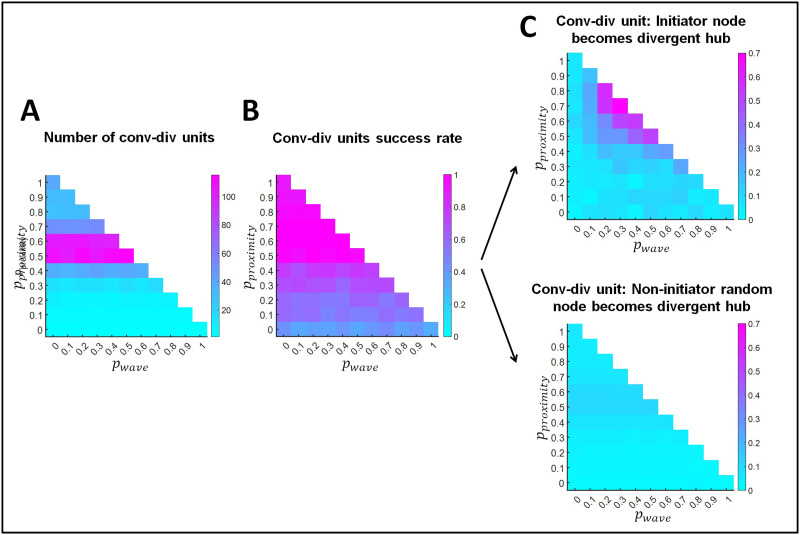
Formation of convergent-divergent units. (A) Total number of convergent-divergent units. (B) Proportion of network instances where at least one convergent-divergent unit exists. (C) Upper panel: Proportion of network instances where a retinal wave initiator node becomes the divergent hub of a convergent-divergent unit. Lower panel: Out of 1,000 random selections of nodes that are not retinal wave initiators, proportion of times that these are a divergent hub of a convergent-divergent unit. Results are shown as a function of the probability of proximity-based rewiring and retinal wave-based rewiring. The remaining probability (i.e., when the sum of the probabilities for proximity-based and retinal wave-based rewiring does not reach 1) corresponds to adaptive rewiring without retinal wave occurrence. For each combination of rewiring rule probabilities (e.g., *p*_*proximity*_ = 0.5, *p*_*wave*_ = 0.2, *p*_*adaptive*_ = 0.3), results are obtained by averaging over 150 networks that evolve from 150 initially random networks.

Now that we have demonstrated the formation of convergent and divergent hubs, we proceed to the formation of convergent-divergent units, which result from the existence of convergent and divergent hubs linked through a direct path. Figure 5A shows the average total number of convergent-divergent units that evolved in the networks. Not surprisingly, its trend is similar to that of convergent and divergent hubs. Figure 5B shows the success rate for the formation of convergent-divergent units. We define this success rate as the proportion of network instances (out of the 150 networks resulting from rewiring) where at least one convergent-divergent unit formed. The success rate increased with larger probabilities for proximity-based rewiring, as also did the connectedness of networks (Figure 2, rightmost panel). That is, the success rate of forming convergent-divergent hubs depends not only on the number of convergent and divergent hubs, but also on the number of direct paths between nodes.

We next considered whether wave initiator nodes were more likely than non-initiator nodes to become the divergent hub of a convergent-divergent unit. Figure 5C (upper panel) indicates that initiator nodes more likely became divergent hubs given a specific combination of probabilities for the rewiring principles (i.e., 0.2–0.5 probability for retinal wave-based rewiring and 0.5–0.8 probability for proximity-based rewiring). Within this range, the probability that a retinal wave initiator node would become a divergent hub increased with the retinal wave-based rewiring probability. The probability that a non-initiator node would become a divergent hub in the convergent-divergent unit was calculated across 1,000 random selections of non-initiator nodes. Importantly, the probability of becoming the divergent hub was larger for retinal wave initiator nodes than for non-initiator nodes in the convergent-divergent unit (see Figure 5C, upper and lower panels).

**Figure 6.**
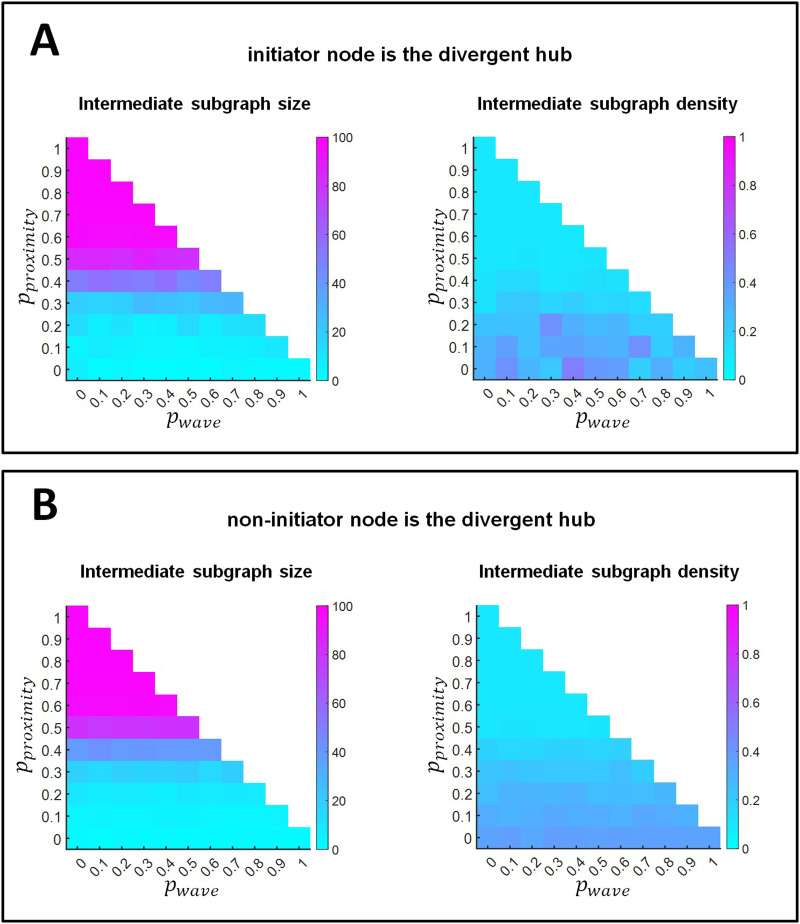
Subgraph structure in convergent-divergent units. (A) Convergent-divergent units where retinal wave initiator nodes are the divergent hubs. (B) Convergent-divergent units where non-retinal wave initiator nodes are the divergent hubs. The left panels represent the size, as number of nodes, of the intermediate subgraphs, and the right panels represent their density. Results are shown as a function of the probability of proximity-based rewiring and retinal wave-based rewiring. The remaining probability (i.e., when the sum of the probabilities for proximity-based and retinal wave-based rewiring does not reach 1) corresponds to adaptive rewiring without retinal wave occurrence. For each combination of rewiring rule probabilities (e.g., *p*_*proximity*_ = 0.5, *p*_*wave*_ = 0.2, *p*_*adaptive*_ = 0.3), results are obtained by averaging over 150 networks that evolve from 150 initially random networks.

In convergent-divergent units, nodes on directed paths between the divergent and the convergent hub are referred to as intermediate nodes. The subgraph integrated by these nodes is called the intermediate subgraph, and processes the information from the convergent hub. The extent to which this subgraph is isolated from the rest of the network determines its processing style and context sensitivity. For intermediate subgraphs containing more than one node, Figure 6 shows their size (i.e., number of nodes, *n*) and density (number of edges, *m*, divided by the maximum number of edges possible: *m*/[*n*(*n* − 1)/2]). We calculated these values both for networks where a retinal wave initiator node was the divergent hub (Figure 6A) and for networks where the divergent hub was a non-initiator node (Figure 6B). In both cases, the size of the intermediate subgraph increased with the probability of proximity-based rewiring. Regarding density, it decreased with increasing proximity-based rewiring probability, until *p*_*proximity*_ > 0.5, where a floor value was reached. This can easily be explained by the fact that in the reported probability regions, all nodes of the graph, excluding the convergent and divergent hubs, were part of the intermediate subgraph.

### Increased Number of In-Links in Nodes Targeted by Retinal Wave Initiator Nodes

**Figure 7.**
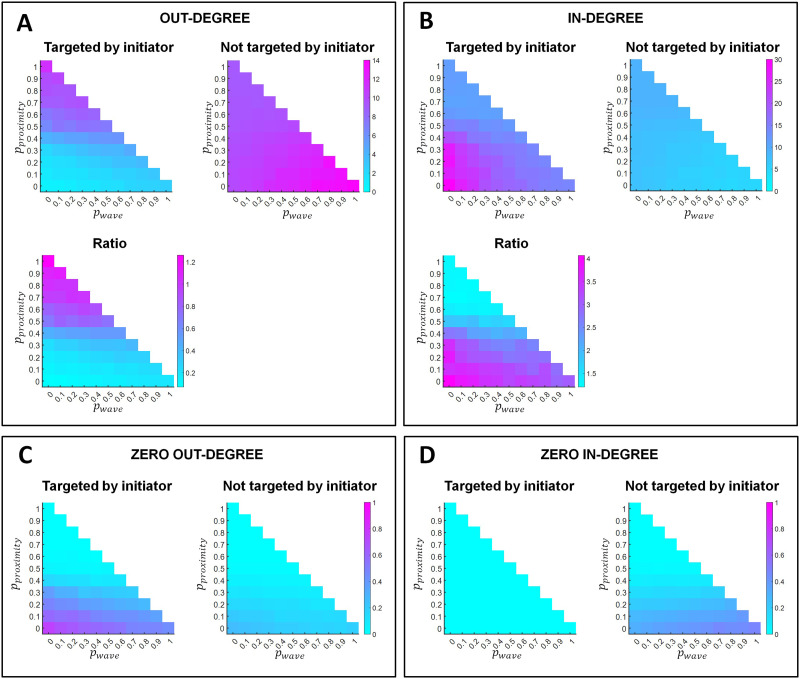
In- and out-connectivity of nodes targeted by the out-links of retinal wave initiator nodes and the rest of non-targeted nodes. (A, B) The left upper panels represent the average number of out-connections (A) and in-connections (B) of nodes targeted by initiators (left panel) and not targeted by initiators (right panel). Note that the vertical scales differ between left and right panels within the top cells. The lower panels represent the ratio between the two upper panels, indicating the probability regions for the different rewiring principles where nodes targeted by initiators most consistently develop a greater number of out-connections (A) and in-connections (B). (C, D) Proportion of nodes with zero out-links (C) and in-links (D) connectivity among those targeted by initiators (left panel) and those not targeted (right panel). Results are shown as a function of the probability of proximity-based rewiring and retinal wave-based rewiring. The remaining probability (i.e., when the sum of the probabilities for proximity-based and retinal wave-based rewiring does not reach 1) corresponds to adaptive rewiring without retinal wave occurrence. For each combination of rewiring rule probabilities (e.g., *p*_*proximity*_ = 0.5, *p*_*wave*_ = 0.2, *p*_*adaptive*_ = 0.3), results are obtained by averaging over 150 networks that evolve from 150 initially random networks.

Interestingly, in the lower panel in Figure 3B we observed that while non-initiator nodes lose outgoing connectivity, they gain incoming connectivity. We might consider this to be an effect of renormalization of the network weights. In that case, the effect would be equally distributed among all non-targeted nodes. However, it rather affects in particular the nodes targeted by the out-links of initiator nodes. Figure 7B shows the in-degree of nodes targeted by the out-connections of retinal wave initiator nodes as well that of non-targeted nodes. The ratio is displayed in the lower panel of Figure 7B. In-link connectivity is generally larger for targeted nodes than for non-targeted ones. The difference is more pronounced at the lower frequencies of retinal wave occurrence. The increase in in-link connectivity is matched with a decrease in the out-connectivity in those nodes (Figure 7A). Among the nodes targeted and not targeted by initiators, Figures 7C and 7D show, respectively, the proportion of nodes that have zero out-connections and zero in-connections. Self-evidently, none of the nodes targeted by initiators in Figure 7D have zero in-degree. However, a significant proportion of non-targeted nodes have zero in-connectivity. Importantly, Figure 7C shows that the proportion of nodes with zero out-link connectivity is larger among nodes targeted by initiators than among non-targeted ones.

The result suggests that the effects of retinal waves on the differentiation of retinal ganglion also affect their direct neighbors. In the retina, the ganglion cells form recurrent circuits with amacrine cells (Xu et al., 2016). Mature amacrine cells are inhibitory and most lack an axon (Masland, 2012). It is possible that the differentiation of their morphology and function are driven by overexpression of Ca^2+^ in these cells, as a result of receiving the retinal wave activity. Intracellular calcium waves regulate the rate of axon extension (Rosenberg & Spitzer, 2011) and, at least in embryonic spinal neurons, the differentiation of inhibitory and excitatory cells. This occurs in a homeostatic manner; that is, when calcium activity is low, more neurons tend to become excitatory, and when it is high, inhibitory (Borodinsky et al., 2004).

**Figure 8.**
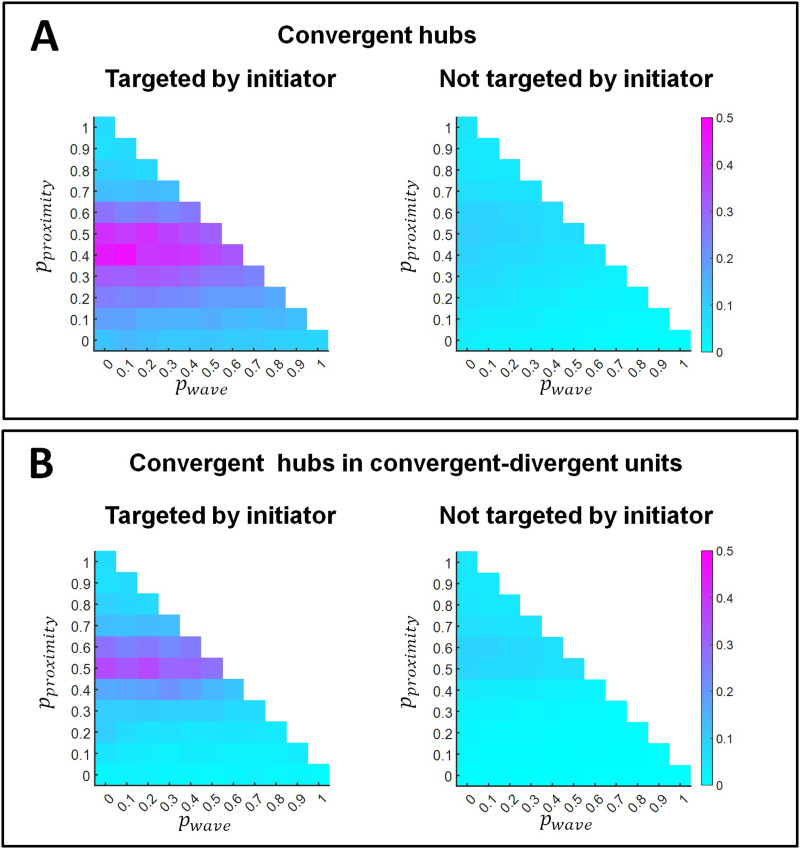
Proportion of convergent hubs (A) and convergent hubs within convergent-divergent units (B) among nodes targeted by the out-links of retinal wave initiator nodes (left panels) and the rest of non-targeted nodes (right panels). Results are shown as a function of the probability of proximity-based rewiring and retinal wave-based rewiring. The remaining probability (i.e., when the sum of the probabilities for proximity-based and retinal wave-based rewiring does not reach 1) corresponds to adaptive rewiring without retinal wave occurrence. For each combination of rewiring rule probabilities (e.g., *p*_*proximity*_ = 0.5, *p*_*wave*_ = 0.2, *p*_*adaptive*_ = 0.3), results are obtained by averaging over 150 networks that evolve from 150 initially random networks.

Finally, we considered whether nodes targeted by initiators were more likely to become convergent hubs than the rest of the non-targeted nodes. Figure 8A shows that the proportion of convergent hubs among targeted nodes is larger than among nodes not targeted by initiators. Furthermore, Figure 8B shows the same for the proportion of convergent hubs within convergent-divergent units. The result suggests that the divergence created by retinal wave activity may prepare the ground for convergence at the level of their axon terminals in LGN (e.g., Hamos et al., 1987; Tavazoie & Reid, 2000) and beyond (Ringach, 2021); that is, RGCs (divergent hubs in a convergent-divergent unit) preferably broadcast their input to convergent hubs in downstream areas (LGN and beyond), thereby reinforcing their role.

## DISCUSSION

Applying a mixture of adaptive and spatial rewiring rules in neural networks with weighted and directed connections results in modular small-world networks with rich-club effect, and convergent-divergent units (Li et al., 2023). Like in the present work, Li et al.’s model relies entirely on spontaneous activity. This activity triggers synaptic plasticity mechanisms that compare well with our adaptive rewiring rules; that is, growth cone behavior at the tip of the axon or dendrite is influenced by the influx of calcium following postsynaptic activation (Jourdain et al., 2003; Kater & Guthrie, 1989, 1990; Kater et al., 1988, 1989, 1990), while spatial rewiring may be thought of as modeling gap junctions.

However, Li et al.’s (2023) model does not have the capacity for sensory processing, a feature for which input systems are required. Considering this limitation, we are interested in how adaptive rewiring can help build the relevant input structures, based on spontaneous activity in the system. We focused on retinal wave activity, in particular on its role in the development of retinal ganglion cells as divergent hubs for projecting retinal activity into the visual system.

Initiator nodes, designated as retinal ganglion cells in an initially random network, received a boost to their outgoing activity, representing the effect of retinal waves. As a result, a significant proportion of initiator nodes developed into divergent hubs. Imposing retinal waves on the system did not interfere with the formation of complex network structures, including the formation of convergent-divergent units (Li et al., 2023; Rentzeperis et al., 2022).

The frequency with which the waves occur parametrically controls the proportion of initiator nodes that develop into divergent hubs. However, the largest proportions occur not with the maximally frequent occurrence of waves, but in an intermediate regime, in accordance with their intermittent occurrence in the developing brain. The reason is that, while initiator nodes increasingly developed outgoing connectivity with the frequency of wave occurrence, this went at the expense of their incoming connectivity. To qualify as divergent hubs, incoming connections are necessary. With very prominent wave activity, initiator nodes increasingly lost their incoming connectivity. This result may suggest new insights into a topic that has been a conundrum in the field of retinal development: A large proportion of all ganglion cells die naturally during development (de Montigny et al., 2023). While it might be intuitive to think that cell death is caused by insufficient input, our results suggest that an overdose of wave activity causes potential ganglion cells to lose their input, and die as a result.

The mechanism that turns initiator nodes into divergent hubs reduces the outgoing connectivity of their direct neighbors. At the same time, these nodes gain incoming connectivity (Figures 3B and 7). This effect may reflect the differentiation of some of these nodes into amacrine cells, which have many incoming connections but no axon. More generally, nodes targeted by initiator nodes are more likely to become convergent hubs (Figure 8A) and feature as such in convergent-divergent units (Figure 8B). This suggests that the retinal wave mechanism that effectuates divergence in the ganglion cells is also responsible for convergence downstream, in LGN and visual cortex. The result suggests that the divergence created by retinal waves could be linked to the formation of convergence in LGN. This is noteworthy, as convergence in LGN (Hamos et al., 1987; Tavazoie & Reid, 2000) and beyond (Ringach, 2021) is well established empirically and is held responsible for the diversity in receptive fields. Its origin has not been well understood. It may be considered a serendipitous effect of our modeling strategy. The current result is limited in several ways. It abstracts from the differentiated ways that remodeling of synapses in the developing brain takes place. It reduces the multistage complexity of retinal wave activity (Maccione et al., 2014) to a single process targeting initiator nodes only. By abstracting from this complexity, we aimed at the discovery of principles, which may play a basic role in more realistic future models.

Another limitation of the model is that it ignores the 3D spatial context and the impact of guidance cues on the navigation of axonal growth cones in network formation. Previous models (Calvo Tapia et al., 2020; Li et al., 2023) offered a complementary third rewiring rule, termed “alignment” (Li et al., 2023) to represent these effects. The “alignment” rule allowed nodes, inter alia, to form chains. The rule was omitted here since it had little impact on the network structure (as opposed to its morphology). In future developments, our model could be combined with the simulation of axonal guidance and diffusive substances in physical space (Bauer et al., 2014). The timing of tectal and thalamic innervation and synapse formation significantly overlaps with the presence of retinal waves (Harada et al., 2007; Lalitha et al., 2020). Our results highlight the possibility that retinal waves promote axonal projections, while relying on the guidance cues to bring them to their destinations, LGN, pretectal nuclei, and superior colliculus. Similar mechanisms in these cell bodies might then arrange for further transport of retinal wave activity.

We conclude that by modeling retinal waves, we have achieved a systematic method to stochastically control the formation of outgoing hubs, or ganglia, in subgraphs of evolving complex networks. Our results suggest that this very basic principle, which drives the formation of divergence in retinal ganglion cells, may also be responsible for the differentiation of amacrine cells and for convergence downstream in the visual system. Ultimately, the retinal waves enable divergence and convergence to arise within the developing visual system.

## CODE AVAILABILITY

The code implementation of the adaptive rewiring algorithm as well as scripts for the analysis of the resulting network topologies and generation of figures are available at https://github.com/raullunadelvalle/Adaptive_Rewiring_CalciumWaves (Luna et al., 2024).

## AUTHOR CONTRIBUTIONS

Raúl Luna del Valle: Data curation; Formal analysis; Funding acquisition; Investigation; Methodology; Software; Visualization; Writing – original draft. Jia Li: Software. Roman Bauer: Visualization; Writing – review & editing. Cees van Leeuwen: Conceptualization; Funding acquisition; Investigation; Methodology; Project administration; Resources; Supervision; Writing – review & editing.

## FUNDING INFORMATION

Cees van Leeuwen, Flemish Organization for Science (FWO), Award ID: Odysseus Grant (G.0003.12.). Raúl Luna del Valle, Boehringer Ingelheim Fonds. Raúl Luna del Valle, MCIN/AEI (https://dx.doi.org/10.13039/501100011033), Award ID: Juan de la Cierva-Formación fellowship (FJC2020-044084-I).

## Supplementary Material



## TECHNICAL TERMS

Spontaneous neural activity: Neural activity that is not triggered by sensory input.
Retinal waves: Spontaneous neural activity that propagates across the developing retina in a wave-like manner.
Adaptive rewiring models: Graph network evolution models where a network topology iteratively changes according to basic neural maturation principles modeled algorithmically.
Small-world network: Network with highly clustered nodes and an average short path length.
Rich-club effect: Tendency of high-degree nodes (i.e., nodes with a large number of in-/out-links) in a network to connect between each other.
Graph: Mathematical representation of a network where a set of nodes (i.e., vertices) are connected through a set of links (i.e., edges).
Convergent-divergent unit: A convergent hub gathers input from local neurons and relays it to isolated units, which feed to a broadcasting divergent hub.
Convergent hub: Node that has at least one out-link and a significant number of in-links that surpass a given threshold.
Divergent hub: Node that has at least one in-link and a significant number of out-links that surpass a given threshold.
Directed graph: Graph in which its edges have a direction, typically indicated by an arrow, which designates the direction of information flow.
In-link: The in-links of a node are the set of edges directed towards it.
Out-link: The out-links of a node are the set of edges originating from it.
Directed path: In a directed graph, a sequence of edges that join two nodes and that are oriented in the same direction.
Modularity: Extent to which a network can be divided into separate node communities or clusters.
Path length: Number of edges within a path linking two nodes.

## Supporting Information

https://github.com/raullunadelvalle/Adaptive_Rewiring_CalciumWaves
