# Supplementary material for: Retinal waves in adaptive rewiring networks orchestrate convergence and divergence in the visual system
Source: Netw Neurosci. 2024 Oct 1;8(3):653–72. doi: 10.1162/netn_a_00370 (PMC11340993; doi:10.1162/netn_a_00370)
Supplement: Supplementary file 1 [file netn-8-3-653-s001.docx]

Supplementary material for:

*“Retinal waves in adaptive rewiring networks orchestrate convergence and divergence in the visual system”*

**Network embeddings**


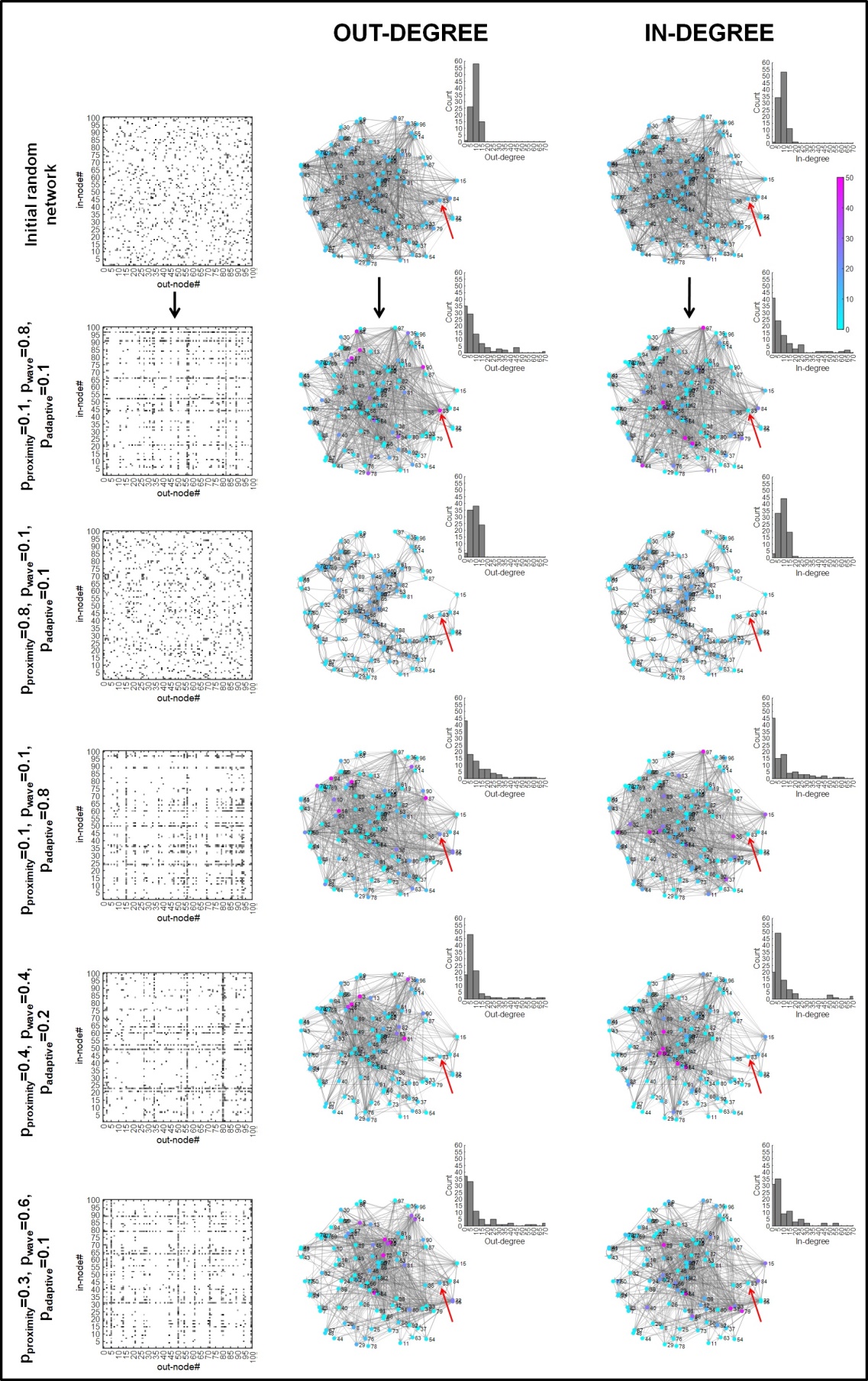


**Figure S1.** Leftmost column: Adjacency matrices of an initially random network (upper plot) and four examples of networks after rewiring using different probabilities for $p_{proximity}$, $p_{wave}$ and $p_{adaptive}$. The adjacency matrices show the in- and out-connectivity of each node, according to its identifier number (node#). The plots to the right show the spatially embedded networks. The central column shows outgoing links, the rightmost incoming links. The numbers next to the nodes indicate the node identifier numbers, and the number of out-connections (OUT-DEGREE) and in-connections (IN-DEGREE) of each is expressed according to the colour bar. The retinal wave initiator is node number 83 (indicated by a red arrow). For each network, the histograms depict the distributions of, respectively, the nodes’ in and out-degrees.
